# The effects of postnatal exposure to low-dose bisphenol-A on activity-dependent plasticity in the mouse sensory cortex

**DOI:** 10.3389/fnana.2014.00117

**Published:** 2014-10-22

**Authors:** Emily A. Kelly, Lisa A. Opanashuk, Ania K. Majewska

**Affiliations:** ^1^Department of Neurobiology and Anatomy, Center for Visual Science, School of Medicine and Dentistry, University of RochesterRochester, NY, USA; ^2^Department of Environmental Medicine, University of RochesterRochester, NY, USA

**Keywords:** bisphenol-A, dendrite, spine, plasticity, ocular dominance, S1, V1

## Abstract

Bisphenol-A (BPA) is a monomer used in the production of polycarbonate plastics, epoxies and resins and is present in many common household objects ranging from water bottles, can linings, baby bottles, and dental resins. BPA exposure has been linked to numerous negative health effects throughout the body, although the mechanisms of BPA action on the developing brain are still poorly understood. In this study, we sought to investigate whether low dose BPA exposure during a developmental phase when brain connectivity is being organized can cause long-term deleterious effects on brain function and plasticity that outlast the BPA exposure. Lactating dams were orally exposed to 25 μg/kg/day of BPA (one half the U.S. Environmental Protection Agency’s 50 μg/kg/day rodent dose reference) or vehicle alone from postnatal day (P)5 to P21. Pups exposed to BPA in their mother’s milk exhibited deficits in activity-dependent plasticity in the visual cortex during the visual critical period (P28). To determine the possible mechanisms underlying BPA action, we used immunohistochemistry to examine histological markers known to impact cortical maturity and developmental plasticity and quantified cortical dendritic spine density, morphology, and dynamics. While we saw no changes in parvalbumin neuron density, myelin basic protein expression or microglial density in BPA-exposed animals, we observed increases in spine density on apical dendrites in cortical layer five neurons but no significant alterations in other morphological parameters. Taken together our results suggest that exposure to very low levels of BPA during a critical period of brain development can have profound consequences for the normal wiring of sensory circuits and their plasticity later in life.

## INTRODUCTION

There is growing public awareness of the possible health impact of exposure to environmental toxicants. One of the most ubiquitous of these is bisphenol-A (BPA), a monomer used in the production of consumer materials such as water and baby bottles (Brede et al., 2003), can linings (Kang et al., 2003), and dental sealants (Suzuki et al., 2000; Joskow et al., 2006). Processes such as heat and pH level shifts are sufficient to trigger BPA migration out of everyday household items and into the surrounding environment (Kang et al., 2003). Emerging evidence indicates that BPA exposure is detrimental to human health; nevertheless, governing bodies continue to debate the interpretation of scientific results. The U.S. Environmental Protection Agency (EPA) has calculated an acceptable daily exposure level that “is likely to be without an appreciable risk of deleterious effects during a lifetime” (U.S. Food and Drug Administration, 2010). However, numerous studies on BPA exposure have described adverse outcomes with much lower doses than the EPA standard (Itoh et al., 2012; Rochester, 2013; Szychowski and Wojtowicz, 2013).

Research on the risks of BPA has focused on the effects of prenatal exposure on the development of the reproductive system; but studies examining brain structure and function suggest widespread negative consequences. Interestingly, BPA exposure during perinatal, postnatal and even during adult periods results in adverse effects on brain processes (Richter et al., 2007) at BPA levels below the current accepted daily exposure level (Hajszan and Leranth, 2010), targeting such mechanisms as synaptogenesis, memory consolidation, and dendritic development. Indeed, BPA has been shown to influence synaptic plasticity in the hippocampus and prefrontal cortex (MacLusky et al., 2005; Eilam-Stock et al., 2012) where the synaptic interface (including the synaptic cleft and presynaptic active zone) have been affected (Xu et al., 2013b). However, our understanding of *how* BPA affects brain development and subsequent function is still in its infancy.

Here, we examine the long lasting effects of low dose BPA exposure during brain development on subsequent activity-dependent remodeling of neural circuits. We used intrinsic signal optical imaging to assess changes in ocular dominance plasticity (ODP) in mice exposed to low-dose BPA during an intense period of synaptogenesis (P5–P21). Our findings show that early BPA exposure results in an attenuation of ODP following 4-day monocular deprivation (4d MD). Our results suggest that even very low dose exposure to BPA during a period of intense synaptogenesis can alter normal development and lead to long lasting changes in the brain.

## MATERIALS AND METHODS

### ANIMALS

Animals were treated in strict accordance with the University of Rochester Committee on Animal Resources and the 2011 NIH Guide for the care and use of laboratory animals. Mice were group housed with food and water available *ad libitum* and were housed under a fixed 12-h light/dark cycle. To prevent any endogenous exposure to BPA, animals were housed in BPA free conditions (Howdeshell et al., 2003). C57BL6 mice (Charles River Laboratories, Wilmington, MA, USA) and mice expressing green fluorescent protein (GFP-M; Feng et al., 2000) were housed in polysulfone cages on alpha-dri paper bedding and fed a phytoestrogen-free diet (2020X; Harlan Laboratories Inc.; USA) and reverse osmosis filtered water. Only glass containers were used for sample preparation and all cages and containers were washed in fresh non-recirculated water. For brain harvesting, mice were anesthetized with sodium pentobarbital (150 mg/kg; i.p.) at P32 and perfused through the aortic arch with ice-cold phosphate-buffered saline [0.1 M PBS, 0.9% NaCl in 50 mM phosphate buffer (pH 7.4)] followed by 4% paraformaldehyde (PFA; in 0.1 M PBS, pH 7.4). Brains were post-fixed in 4% PFA for 2 h and transferred to an increasing gradient of sucrose (10, 20, 30% in ultra-pure water) at 4°C. Brains were sectioned coronally at a 50 μm thickness on a freezing, sliding microtome. Both female and male mice were included in the study. No significant differences were observed between sexes in any of the analyses and data from both sexes were pooled. See **Table 1** for all animal numbers.

**Table 1:** Animal and tissue used.

Experiment	Treatment	*n*	Sections/animal	Spines	Figure
Optical imaging	NoMD oil	4			1
	4dMD oil	6			1
	NoMD BPA	6			1
	4dMD BPA early	6			1
	4dMD BPA late	5			1
Parvalbumin	Oil	5	4		2
	BPA	11	4		2
Cortical thickness	Oil	5	4		2
	BPA	9	4		2
Myelin basic protein	Oil	5	6		3
	BPA	5	6		3
Dendritic spine density	Oil	6	3	230	4
	BPA	6	3	375	4
Dendritic spine morphology	Oil	6	3	230	5
	BPA	6	3	375	5
Dendritic spine dynamics	Oil	3		365	6
	BPA	3		331	6
Microglial density analysis	Oil	4	3		7
	BPA	5	3		7
Microglial markers	Oil	3	4		8
	BPA	3	4		8

### BPA EXPOSURE

**FIGURE 1:**
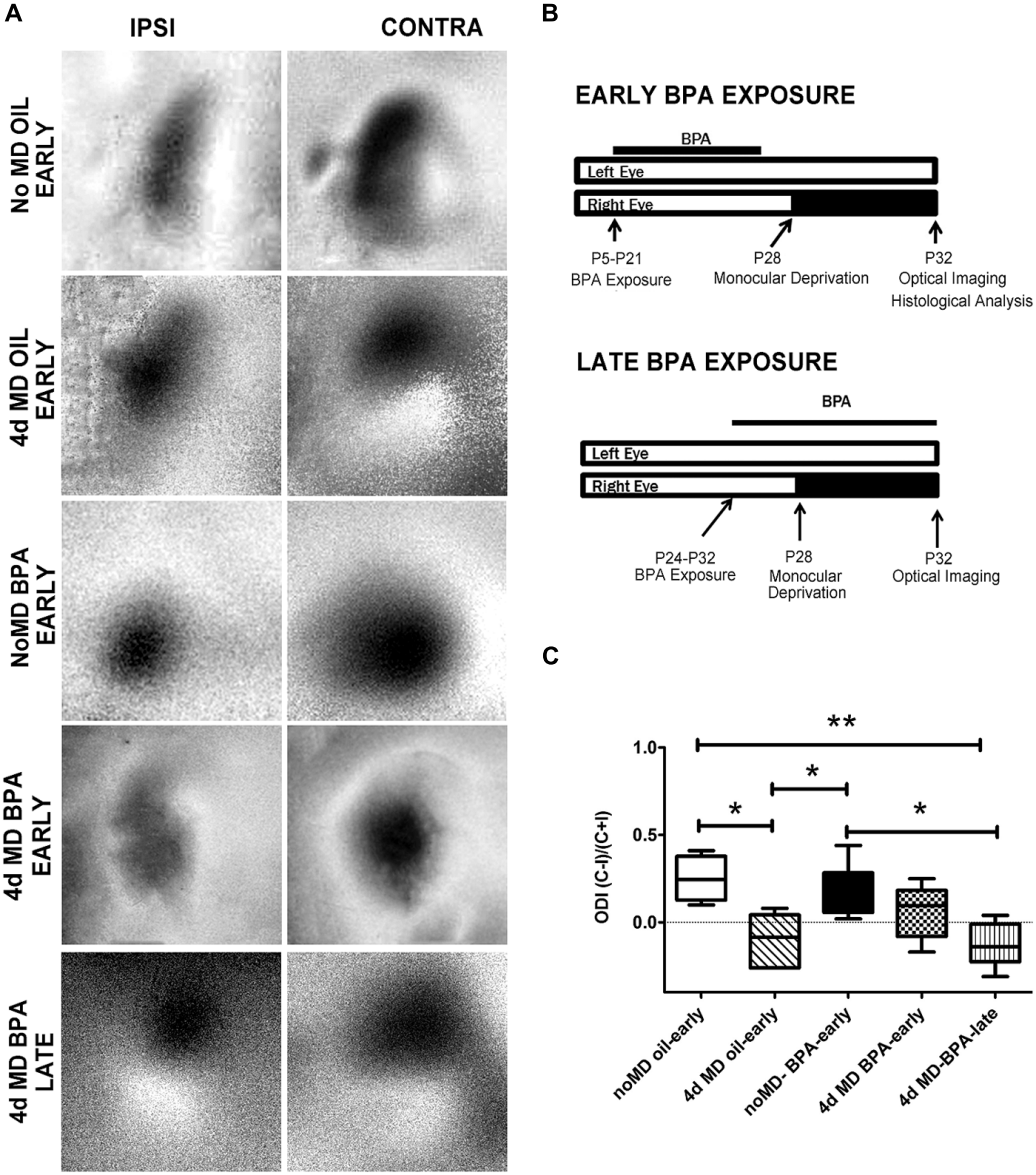
**Ocular dominance plasticity is attenuated in BPA-exposed mice. (A)** Representative images of amplitude maps generated following intrinsic signal optical imaging (iOS). Non-deprived mice (No MD-early) showed an expected contralateral bias (strong contralateral amplitude when compared to ipsilateral amplitude; top panel). Following 4-days of monocular deprivation (4d MD-early), there was a shift in responsiveness toward the ipsilateral eye. NoMD BPA-early treated mice showed similar amplitude maps as NoMD Oil-early mice. Following 4d MD in early exposure BPA mice (4d MD BPA-early), maps retain a contralateral bias. A normal OD shift was observed after 4d MD in late exposure mice (4d MD BPA-late). **(B)** Treatment paradigms for *early exposure* (top schematic) and *late-exposure* BPA mice. **(C)** Ocular dominance index (ODI) results for BPA and Oil treated conditions. Values above 0 represent a contralateral ocular dominance bias (expected result in normal seeing mice). Values below 0 suggest an ipsilateral bias (expected result following monocular deprivation during the critical period). *p < 0.05; **p < 0.01.

Animals were exposed to BPA at two different time points; during an early period of cortical development [*early exposure*; postnatal day (P)5–P21; No MD BPA-early, 4d MD BPA-early, No MD Oil-early, and 4d MD Oil-early; **Figure 1C**] and a later period of cortical development (*late exposure;* P24–P32; critical period for rodent visual cortical plasticity; 4d MD BPA-late; **Figure 1C**). For the *early exposure* paradigm, lactating mouse dams were fed a 0.1 *g* portion of a vanilla wafer cookie (Back to Nature Foods Company; Madison, WI, USA) containing either BPA (25 μg/kg body weight/day; Sigma Aldrich CAT# 239658) dissolved in tocopherol-stripped corn oil (Sigma Aldrich CAT# C8267) or corn oil alone and pipetted onto the cookie at approximately the same time each day. For the *late exposure* paradigm, cookies containing BPA or oil were fed directly to weaned mice. Thus both the ingested dose and the route of BPA delivery differed across the *early* vs. *late* exposure groups (mothers milk vs. direct consumption).

### INTRINSIC SIGNAL OPTICAL IMAGING

To examine the extent of ODP after BPA exposure, mice were monocularly deprived for 4 days at the height of the critical period for cortical plasticity. Intrinsic signal optical imaging was performed using a DALSA 2M30 CCD camera (Kalatsky and Stryker, 2003). On P28 ± 2, lid margins were resected and lids sutured under isoflurane anesthesia (2–3%). After 4 days of MD, animals were anesthetized with isoflurane (2–3%) along with chlorprothixene (2 mg/kg) and the sutures removed for imaging. The skull over visual cortex was cleared, covered with agarose (1%) and a coverslip and illuminated with 700 nm light. Anesthetic level was maintained with isoflurane (0.75%) during imaging. An image of the vascular pattern was obtained through the skull by illumination with a green filter (550 nm). Intrinsic signal images were then captured using a red filter (700 nm). Visual stimuli consisting of white horizontal square-wave bars on a neutral background moving downward (270°) and upward (90°) for 6 min per run, were presented to each eye separately. The amplitude of the fast fourier transform component in the binocular visual cortex was analyzed oﬄine using Matlab to determine ocular dominance (OD; Kalatsky and Stryker, 2003; Tropea et al., 2010). OD was compared between BPA exposed animals and oil exposed controls. An ocular dominance index (ODI) was calculated as (contralateral - ipsilateral)/(contralateral + ipsilateral) based on the average pixel intensities of the images obtained during visual stimulation of each eye. Positive ODI values indicate a contralateral bias; negative values indicate an ipsilateral bias.

### IMMUNOHISTOCHEMISTRY

Fixed brain sections containing visual cortex were immersed in 0.1% sodium borohydride (in 0.1 M PBS) for 30 min at room temperature (RT), washed in 0.1 M PBS, and processed freely floating. Sections were blocked in a solution containing 0.5% bovine serum albuminin (BSA), 5% normal serum and 0.3% Triton-x for 2-h. Sections were then incubated for 24–48 h in either rabbit anti- Iba-1 [ionized calcium binding adaptor molecule-1; microglia- marker; 1:1000; Wako; (Imai et al., 1996)], or mouse anti-MBP [myelin basic protein (MBP); 1:1,000; Covance SMI-94]. Next, sections were incubated for 2-h in a solution containing either biotinylated goat anti-rabbit IgG or biotinylated goat anti-mouse IgG, respectively. Specific activity was detected using an ABC reagent (1:100; Vector Laboratories Inc, Burlington CA) and visualized with 3,3-diaminobenzidine (0.5 mg/ml) and hydrogen peroxide (0.03%) in buffer solution (DAB peroxidase kit; Vector Laboratories). Sections processed for fluorescent immunoreactivity were first preincubated in a blocking solution (as detailed above) and then incubated for 48-h in rabbit anti-parvalbumin [parvalbumin (PV);1:10,000; PA1-933; Affinity Bio Reagents], rat anti-MHC II (I-A/I-E; 1:5,000; Cat# 556999; BD Pharmingen), rabbit anti-Iba-1, or mouse anti-CD68 (ED-1; 1:800, ab31630; Abcam). Sections were washed in 0.1 M PBS and incubated for 2-h in a solution containing anti-rabbit Alexa 594 or 647 or anti-mouse Alexa 488 or anti-rat Alexa 488 (Invitrogen). Sections for light microscopy were mounted onto a microscope slide from a 1% gelatin solution (in 99% ethanol), dehydrated in ascending concentrations of ethanol, cleared in xylene and coverslipped with DPX mounting media (Electron Microscopy Sciences). Sections treated with immunofluorescence were coverslipped with Prolong Gold (Invitrogen) antifade media.

Epifluorescence microscopy images were taken on a BX51 Olympus scope at X 10 magnification (UPlanFL N; X 10/0.30; Olympus, Tokyo, Japan) and X 20 magnification (UPlanFL N; X 20/0.50; Olympus) mounted with a Spot Pursuit RT color digital camera (Diagnostic Instruments, Sterling Heights, MI, USA). Following image acquisition, images were analyzed using Image J software (freeware: http://rsb.info.nih.gov/ij/). For cell density analysis, all immunoreactive cell bodies were counted by hand, the cortical area was measured and densities were imported into Prism (Graphpad Software, Inc; La Jolla, CA, USA) for statistical analysis. For staining intensity analysis, the image was thresholded to encompass all immunoreactive regions. The intensity of pixels within the thresholded area was measured. To determine the laminar specific changes in MBP immunoreactivity, images were imported into Image J. Background intensity values were obtained from unstained portions of layer 1 within each image and subtracted from density averages obtained for each layer. Laminar densities were measured using the profile function of Image J as detailed by (McGee et al., 2005). Cortical thickness was assayed in sections from comparable rostro-caudal locations using the line tool in Image J.

### DENDRITIC SPINE ANALYSIS

Green fluorescent protein-M mice were subject to *early exposure* of BPA or Oil as described above. Animals were perfused between P32 and P35. Brains were sectioned on a freezing sliding microtome to a 50 μm thickness. Sections were mounted out of a 0.1 M PBS solution and coverslipped with Prolong Gold (Invitrogen) antifade media. Confocal microscopy image acquisition and spine analysis was performed per (Bogart et al., 2011). Briefly, layers 2/3 within the primary somatosensory cortex (S1) were identified for imaging on a Zeiss LSM 510 confocal microscope (Care Zeiss, Thornwood, NY, USA). The distributions of imaged areas within S1 were similar between experimental conditions. GFP- labeled brain sections were excited at 488 nm and imaged through an HFT 514/633 dichroic and 530–600 nm band pass filter. Excitation power and settings for pinhole and detector gain were optimized to minimize photobleaching and utilize the full dynamic range of fluorophore emission intensity. High resolution (512 × 512 pixels) confocal image stacks of layer 5 apical dendritic branches located in layer 2/3 were collected using a 100x oil-immersion lens (NA 1.46), at a digital zoom factor 2 (pixel size 0.082 μm), and a z-step of 0.5 μm. Additional z-stacks were collected using lower power objectives to document the position of acquired images within the dendritic arbor stacks. Dendritic segments of the primary apical dendrite in layer 2/3 were located between 70 and 150 μm from the pial surface and were selected based on the quality of GFP expression and resulting signal-to-noise ratio, so that spines could be identified and measured as accurately as possible. Dendritic diameters were not statistically significantly different between groups (OIL: 1.65 ± 0.13 μm; BPA: 1.68 ± 0.13 μm; Student *t*-test; *p* > 0.05).

Following image acquisition, z-stacks were exported to TIF format using Zeiss’s Axiovision software (release 4.6). Image analysis was then done using Image J. To quantify spine density, spines were identified by manually stepping through the z-stack, and definite spines were marked on the projected image. Specifically, only spines located in plane with their parent dendrite branch were marked and counted. Spines falling out of plane and those projecting from the parent dendritic branch in solely the z-dimension were systematically excluded from our counts even if they were visually identifiable as spines. Please note that for the purposes of this study we define spines as all visible dendritic protrusions and filopodia are included in the analysis. After all spines on a segment were marked, segment length was measured using the segmented line tool. 3D segment length was accounted for by measuring the absolute difference in depth between the two ends of the segment and using Pythagoras’ theorem. Spine density was then computed as the number of spines per micron of dendrite. We also analyzed the dimensions of dendritic spines. Spine length was measured on maximum intensity projections using a segmented line tool to draw a line from the most distal point of the spine head to the base of the spine neck where it connects to the parent dendritic branch. Measurements of spine head and neck width were made based on fluorescence measurements. The fluorescence profile of a line placed along the center of the head and neck was determined and fit to Gaussian using custom-written algorithms in MATLAB (The MathWorks, Inc., Natick, MA, USA). The full-width half-max was taken as a measure of spine head width. This method may overestimate the size of small spines that fall under the limit of the resolution of our confocal microscope. The amplitude of the Gaussian fit to the spine neck fluorescence profile was normalized to the amplitude of the fit to the spine head profile as a relative measure of spine neck width. Background fluorescence was subtracted before fitting on a dendrite-by-dendrite basis. Great care was taken to avoid saturation in images, and saturated points were removed from the fluorescence profiles. Spines with more than two saturation points were removed from the analysis as it was determined that accurate fits were obtained if fewer than three points were omitted. This affected <2% of the population of spines.

### TWO-PHOTON IMAGING AND DENDRITIC SPINE TURNOVER ANALYSIS

For two-photon imaging, mice were anesthetized with a fentanyl cocktail (fentanyl; 0.05 mg/kg of body weight; midazolam; 5 mg/kg; metatomadin; 0.5 mg/kg; i.p.); the skull was exposed, cleaned and glued to a thin metal plate. Primary somatosensory cortex (S1) was identified according to stereological coordinates. The skull above the imaged area was thinned with a dental drill. During surgery and imaging, the animal’s temperature was kept constant with a heating pad and anesthesia was maintained with periodic administration of fentanyl. Imaging and data analysis were carried out as previously described (Majewska et al., 2006). A custom-made two-photon scanning microscope (Majewska et al., 2000) was employed, using a wavelength of 920 nm and a 20x 0.95 NA objective lens (Olympus, Melville, NY, USA) at 8.5x digital zoom. A map of the blood vessels was taken as a reference point. After image acquisition the animal’s scalp was sutured and the animal was allowed to recover before being placed back in its home cage. Four days later, the animal was re-anesthetized and the skull re-exposed. The blood vessels map and dendritic architecture were used to identify the same imaging regions. Dendritic protrusions were identified as persistent if they were located within 0.5 μm laterally on the subsequent imaging session. Elimination and formation rates refer to the numbers of new spines and lost spines, respectively, observed on the second imaging time point divided by the total number of spines present in the first imaging session.

### LIPOPOLYSACCHARIDE (LPS) CRANIAL INJECTIONS

As a positive control for microglial activations, we performed intracranial lipopolysaccharide (LPS) injections (Chugh et al., 2013) to induce focal inflammation in the brain (*n* = 3, C57B/6 mice, P32). Mice were anesthetized with a fentanyl cocktail (fentanyl; 0.05 mg/kg of body weight; midazolam; 5 mg/kg; metatomadin; 0.5 mg/kg; i.p.) and placed in a stereotaxic frame. The top of the head was swabbed with 70% ethanol and betadine before the skull was exposed. The surface of the skull was cleared and the injection coordinates (A/P -1.58, D/L +2.5) were calculated from bregma using The Mouse Brain atlas (Franklin and Paxinos, Third Edition). 2.5 mg/ml LPS (in saline, 1 ul total volume) was injected over 5 min in the left hemisphere. Following injections, the scalp was sutured and the animal was allowed to recover before being placed back in its home cage. Animals were sacrificed 24-h later (as described above) and the brains were harvested and sectioned for histological staining.

### STATISTICAL ANALYSIS

Statistical analysis was performed using Prism (Graphpad Software, Inc; La Jolla, CA, USA). Significance was determined using two-tailed Mann Whitney test for comparison between two groups. For multi-group comparison, one-way ANOVA and Bonferroni *post hoc* test (for those data where the *p-*value of the ANOVA was <0.05) were performed to determine significance level. All data are reported as mean ± SEM.

## RESULTS

### OCULAR DOMINANCE PLASTICITY IS ATTENUATED IN MICE EXPOSED TO BPA EARLY IN DEVELOPMENT

While several studies have linked BPA exposure with changes in brain development and function, there is still a lack of understanding of how BPA exposure affects plastic processes that drive the remodeling of neural networks throughout life. We therefore set out to examine whether early life low-dose exposure to BPA would disrupt cortical synaptic plasticity. We chose to focus on a well described form of activity-dependent plasticity which is robustly elicited in the rodent binocular visual cortex following 4d MD during the visual critical period (Hubel and Wiesel, 1970). Under normal conditions (in the absence of visual manipulation; No MD Oil-early), the cortical response to visual stimulation of the contralateral eye is inherently higher than that of the ipsilateral eye resulting in a binocular response that is contralaterally biased (**Figure 1A**, No MD Oil-early). 4d MD during the visual critical period (∼P28) results in a reduction of the response to visual stimulation of the now deprived contralateral eye and a subsequent increase in the response in the non-deprived ipsilateral eye (**Figure 1A**, 4d MD Oil-early). The resulting experience-dependent shift in responsiveness away from the contralateral eye is called ODP.

To determine how BPA exposure affects cortical synaptic plasticity, lactating dams were fed a vanilla cookie impregnated with either 25 μg/kg/day BPA or oil daily during the period that their pups were postnatal day 5 (P5) to P21. Animals had their contralateral eye monocular deprived on P28 and were assayed for ODP using intrinsic signal imaging on P32 (**Figure 1A**; No MD BPA-early, 4d MD BPA-early; **Figure 1B**, top panel). Notice that in this *early exposure paradigm*, the animals experience BPA during a developmental window which encompasses a period of rapid synaptogenesis and circuitry development, but that exposure ends a week before plasticity is induced.

To compare the difference in deprived and non-deprived eye responses across animals, an ODI was generated based on the response magnitudes obtained for each eye within the binocular visual cortex (**Figure 1C**; see methods). An ODI of -1 indicates that the animal’s binocular zone responds only to stimulation of the ipsilateral eye, an ODI of +1 indicates responses to only the contralateral eye. An ODI of 0 would mean that the binocular zone responds equally to stimulation of the ipsilateral or contralateral eye. In normal development, MD elicits a shift from positive (contralaterally biased) toward negative ODI values indicative of ODP. Non-MD animals (no MD-oil early) imaged at P32 demonstrated a typical contralateral bias (**Figure 1C**, open bar). Consistent with previous findings, 4d MD resulted in a shift in OD toward the ipsilateral non-deprived eye in MD-oil control mice (**Figure 1C**, 4d MD Oil-early, diagonal filled bar). Results from animals treated with an early low-dose of BPA but not deprived were similar to control mice (**Figure 1C**, No MD BPA-early, solid bar). However, animals treated with BPA early during development (P5–P21), showed a reduced shift toward the ipsilateral eye following 4d MD which was not statistically significantly different from the ODI of non-MD animals (**Figure 1C**, 4d MD-BPA early checkerbox-filled bar), suggesting that ODP was impaired.

We therefore wondered if BPA could also affect plasticity acutely or whether its effects were limited to a specific developmental period. Therefore, we repeated the experiment in the *late exposure* group, where we sought to determine if exposing animals to BPA only during the critical period of visual cortical plasticity and development would have similar effects. Animals were fed vanilla cookies containing BPA or oil from P24-P32 and were assayed on P32 (**Figure 1B**, lower panel). In contrast to early exposure animals (4d MD-BPA-early), animals exposed to low-dose BPA during the critical period MD (4d MD-BPA-late), showed a significant shift in ODI indicative of normal ODP (**Figure 1C**, 4d MD-BPA-late, vertical line-filled bar). It is important to note however, that the doses were not the same for the early and late group due to the delivery route (lactation vs. direct ingestion, respectively). While this result may suggest that low dose exposure to BPA can affect the long term plasticity of the brain, but that its effects are limited to critical periods of brain development, more work is needed to fully understand how the dose and timing of BPA exposure affect plasticity.

### PARVALBUMIN NEURON DENSITY AND MYELIN BASIC PROTEIN EXPRESSION ARE NOT AFFECTED BY BPA EXPOSURE

Ocular dominance plasticity requires the initial maturation of cortical circuitry and the regulated expression of specific cellular and network properties (Hensch, 2005). Both inhibitory neuron maturation and myelination play a considerable role in cortical development and are critical regulators of ODP. We sought to determine if low-dose BPA exposure during early development affects the maturation of cortical inhibitory networks and myelination which could in turn dampen plasticity during the visual critical period.

**FIGURE 2:**
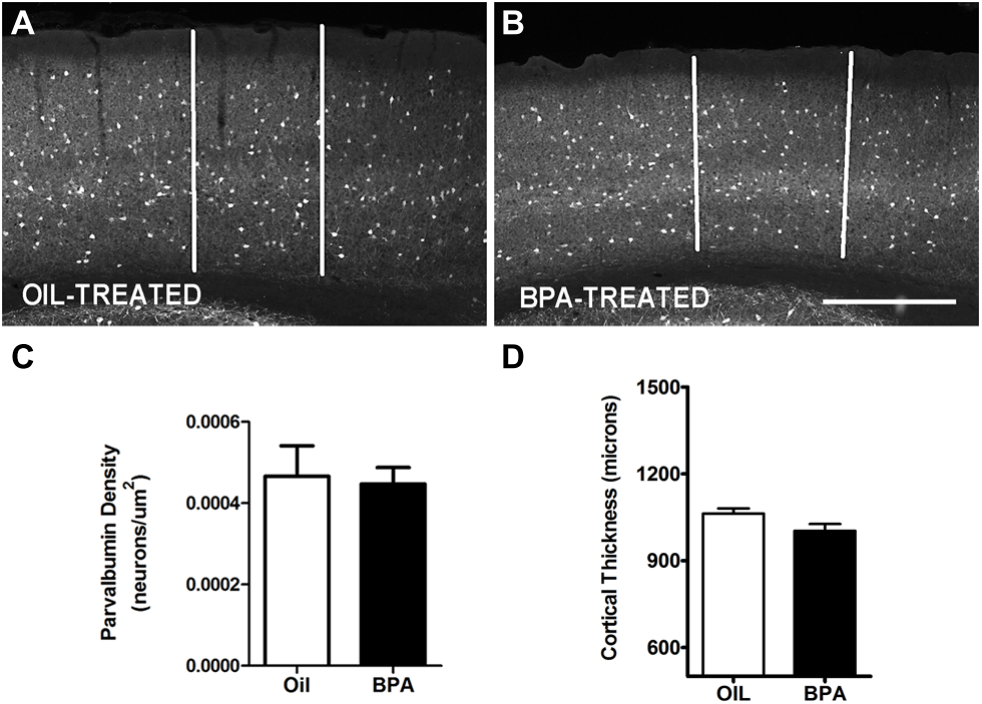
**Parvalbumin expression is unchanged in BPA exposed mice.** Representative examples of anti-parvalbumin immunoreactivity in mice treated with oil **(A)** or low-dose BPA **(B)**. Notice that PV cells are distributed throughout the cortical layers. White vertical bars demarcate the binocular visual cortex, in the coronal plane, where measurements were taken. **(C)** Quantitative analysis of parvalbumin immuno-labeled cell density in the binocular visual cortex. **(D)** Cortical thickness measurement in oil-treated and BPA-treated mice. Scale bar = 200 μm.

Parvalbumin is a calcium binding protein that co-localizes with GABAergic interneurons (Celio, 1986) and has previously been shown to be regulated during periods of synaptic plasticity (Cellerino et al., 1992). We performed immunofluorescent histochemistry and quantified anti-PV reactivity in the mouse visual cortex following *early exposure* to oil or low-dose BPA. PV-reactive cells were distributed in all cortical layers in both oil and BPA treated animals (**Figures 2A,B**). We found no statistically significant difference in PV cell density in binocular visual cortex between the two conditions (**Figure 2C**). Since cortical thickness can affect cell packing density and contribute to attenuation or inflation in cell counts, and BPA may affect overall brain development, we next sought to determine if cortical thickness was altered in BPA-treated mice. Comparable rostro-caudal regions of the visual cortex were measured in both the Oil-treated and BPA-treated mice. We found no significant differences in cortical thickness between the two groups (**Figure 2D**).

**FIGURE 3:**
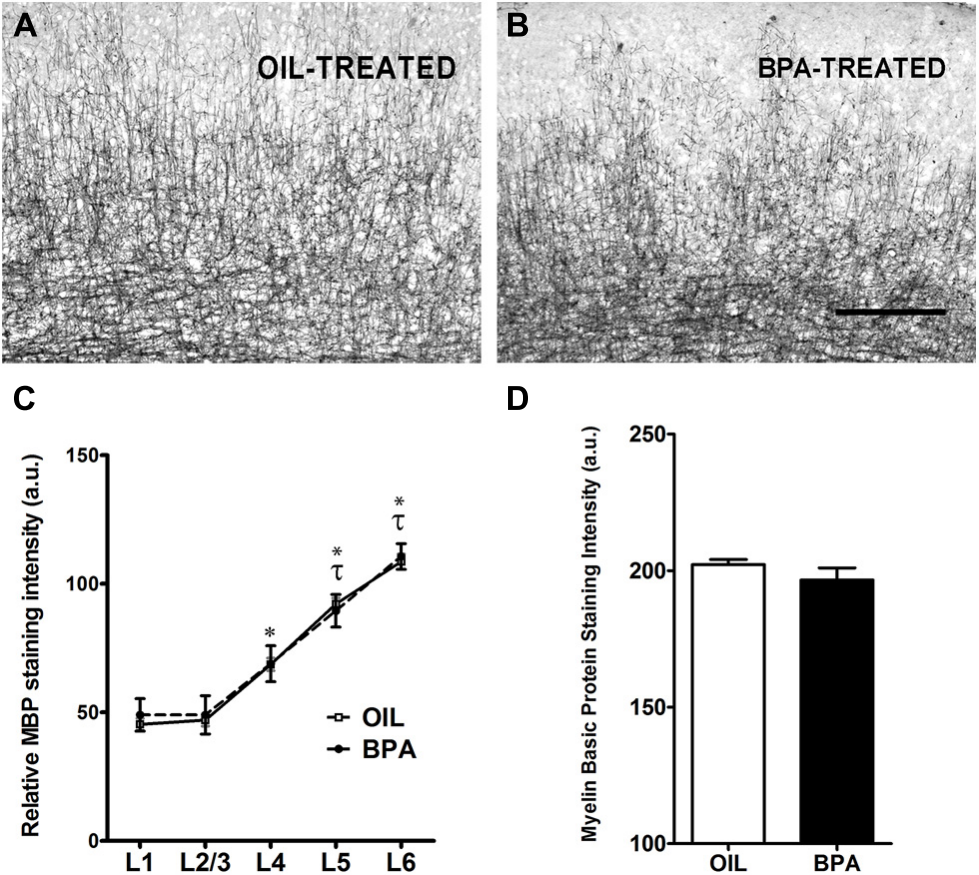
**Myelin basic protein expression is unchanged in BPA exposed mice.** Representative examples of anti-myelin basic protein immunoreactivity in the binocular visual cortex of mice treated with oil **(A)** or low-dose BPA **(B)**. **(C)** Quantification of relative MBP staining intensity across cortical layers shows no significant differences across treatment conditions (Oil-treated, open circles; BPA-treated, closed circles). Asterisk denotes significance across layers *within* the oil-treated group. Specifically, layers 4–6 in the oil-treated groups were significantly different from their neighboring layers as well as from layers 1–3. Tau denotes significance across layers *within* the BPA-treated groups, where only layers 5–6 were significantly different from other layers. **(D)** Quantitative analysis of myelin basic protein immuno-staining intensity throughout all cortical layers. No significant differences were observed between BPA and oil treated animals. Scale bar = 100 μm.

Myelin basic protein (MBP) is a compact myelin marker which is developmentally up-regulated in a layer specific manner. MBP up-regulation is thought to contribute to the closure (inhibition) of critical period plasticity in the rodent cortex (McGee et al., 2005). To determine whether MBP expression was regulated by early exposure to BPA, oil and BPA-exposed brain tissue was immunohistochemically stained with an antibody against MBP. Low magnification images showed dense myelin fiber staining in layer 6 of the visual cortex, which gradually declined in superficial layers in both experimental conditions (**Figure 3A**, oil-treated; **Figure 3B**, BPA-treated). Lamina-specific analysis of MBP immuno-reactivity showed no significant difference between experimental conditions across layers (**Figure 3C**; Oil-treated, open circles; BPA-treated, filled circles). As expected, MBP immunoreactivity was significantly denser in infragranular layers within experimental conditions, showing significantly more reactivity in Layers 4, 5, and 6 in both oil and BPA-treated animals. When all layers were pooled, we found no significant difference in anti-MBP immunoreactivity following BPA exposure (**Figure 3D**).

### LOW-DOSE BPA EXPOSURE AFFECTS DENDRITIC SPINE DENSITY

**FIGURE 4:**
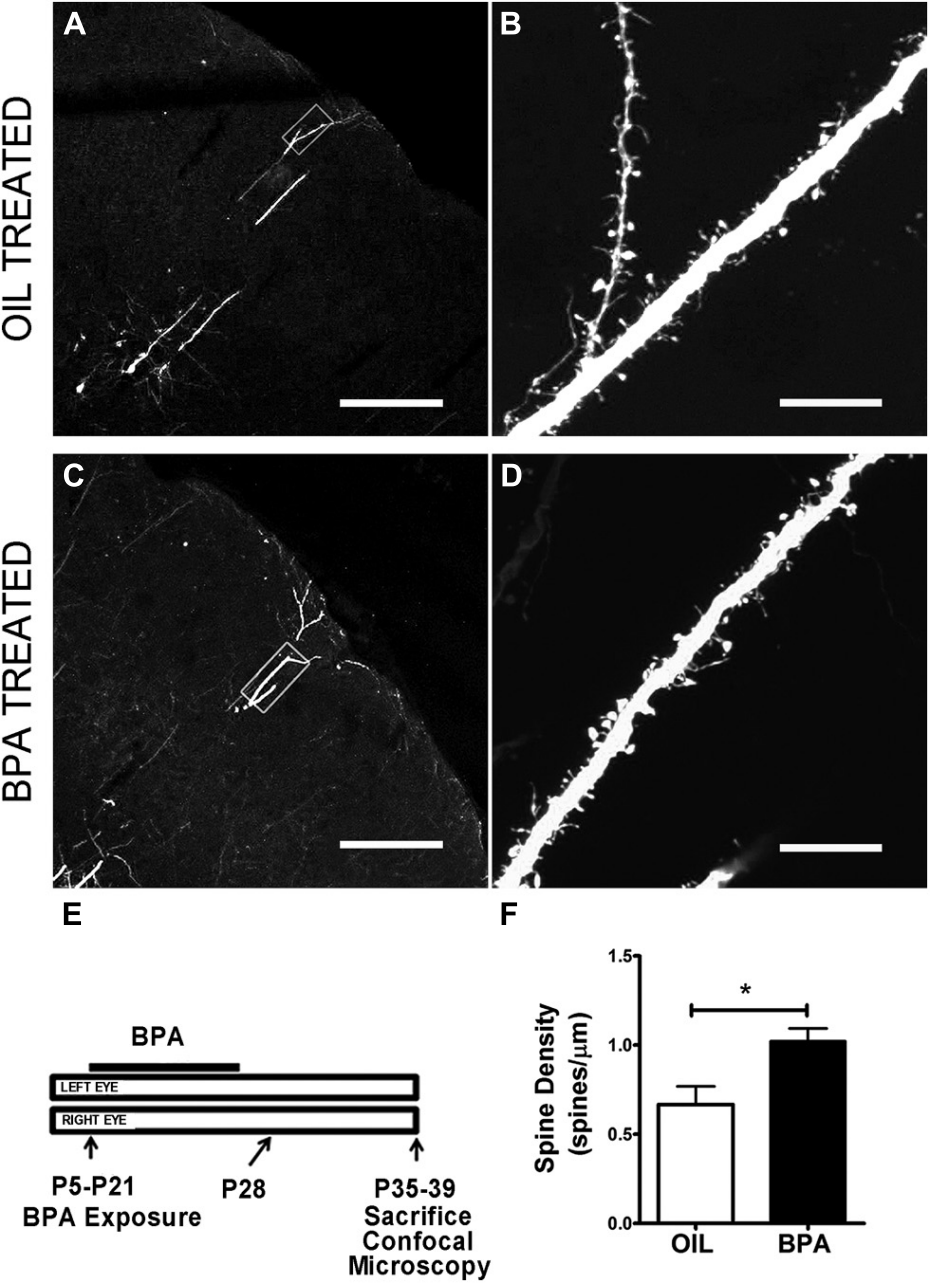
**Dendritic spine density is increased in BPA exposed mice.** Confocal images showing representative examples of apical dendrites of layer 5 neurons in somatosensory cortex at low magnification following oil **(A)** and BPA treatment **(C)**. High magnification images of boxed regions in **(A)** and **(C)** showing distribution and qualitative appearance of dendritic spines in oil **(B)** and BPA treated animals **(D)**. **(E)** Schematic showing exposure parameters for BPA treatment. **(F)** Quantitative analysis showing that BPA exposure during the early postnatal period increases dendritic spine density on apical dendrites of layer 5 neurons (solid bar) relative to oil control (clear bar). *p < 0.05. Scale bars: **A,C** = 200 μm; **B,D** = 10 μm.

Functional synaptic changes during plasticity are accompanied by structural changes at the level of the dendritic spine. We wondered whether low dose BPA exposure early in development affected the maturation of dendritic spines which could affect the implementation of later synaptic changes. To investigate the effects of low-dose BPA exposure on dendritic spine morphology, we used confocal microscopy to analyze fixed sections of somatosensory cortex from GFP-M animals which express GFP in subsets of cortical neurons (**Figures 4A–D**). Animals were treated with a low-dose of BPA from postnatal day 5 (P5) to P21 and sacrificed between P35 and P39 for microscopy analysis (**Figure 4E**). Primary apical dendritic branches of layer 5 cortical neurons residing in layer 2/3 were imaged at high magnification and dendritic spines were counted. Quantitative analysis showed a significant increase in dendritic spine density in animals treated with BPA, as compared to mice treated with oil (**Figure 4F**, oil = 0.667 spines/μm; BPA = 1.022 spines/μm; *p* < 0.05; Student’s *t*-test).

**FIGURE 5:**
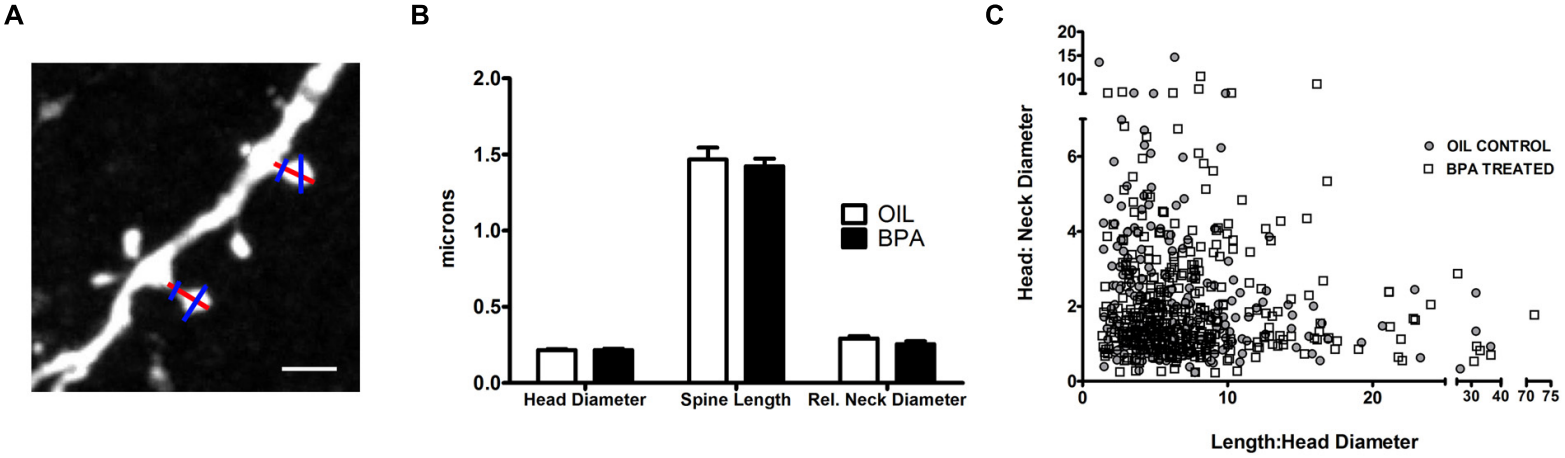
**Quantitative analysis of dendritic spine morphological changes in BPA exposed mice. (A)** A representative high magnification confocal image of dendritic spines on apical dendrites of layer 5 neurons. Lines indicated measurements of dendritic spine parameters. Spine Length measurements (tip of the spine to its base at the dendrite, red line); Spine head diameter (measured at the widest point of spine, blue line) and relative neck width (blue line across neck). Scale bar = 2.5 μm. **(B)** Morphological measurements of dendritic spines, including spine head diameter, spine length and relative neck diameter, were similar for oil treated (clear bar) vs. BPA treated (solid bar) mice. **(C)** Spines can be loosely classified into morphological “types” by comparing their relative head, neck, and length dimensions. The complete overlap of spines from the two conditions indicates that BPA exposure does not elicit a change in spine type distribution (oil = filled in circle, BPA = open square).

Next, we sought to determine if BPA induced morphological changes in dendritic spines (**Figure 5A**). No significant differences in spine head diameter, spine length or relative neck diameter were found between oil and BPA-treated animals (**Figure 5B**). Comparison of spine morphological parameters can be used to determine the developmental profile of dendritic spines. For example, a high head:neck ratio would indicate a mushroom headed (mature) spine, while a low spine head:neck and spine length:head diameter might indicate a more immature phenotype. For both oil and BPA-treated animals, spines appeared to exist as a morphological continuum rather than fall into distinct morphological classes. Furthermore, the distribution of spine morphological parameters overlapped extensively between the two conditions, suggesting that the morphological distribution of dendritic spines in BPA-exposed animals is similar to that in controls (**Figure 5C**).

**FIGURE 6:**
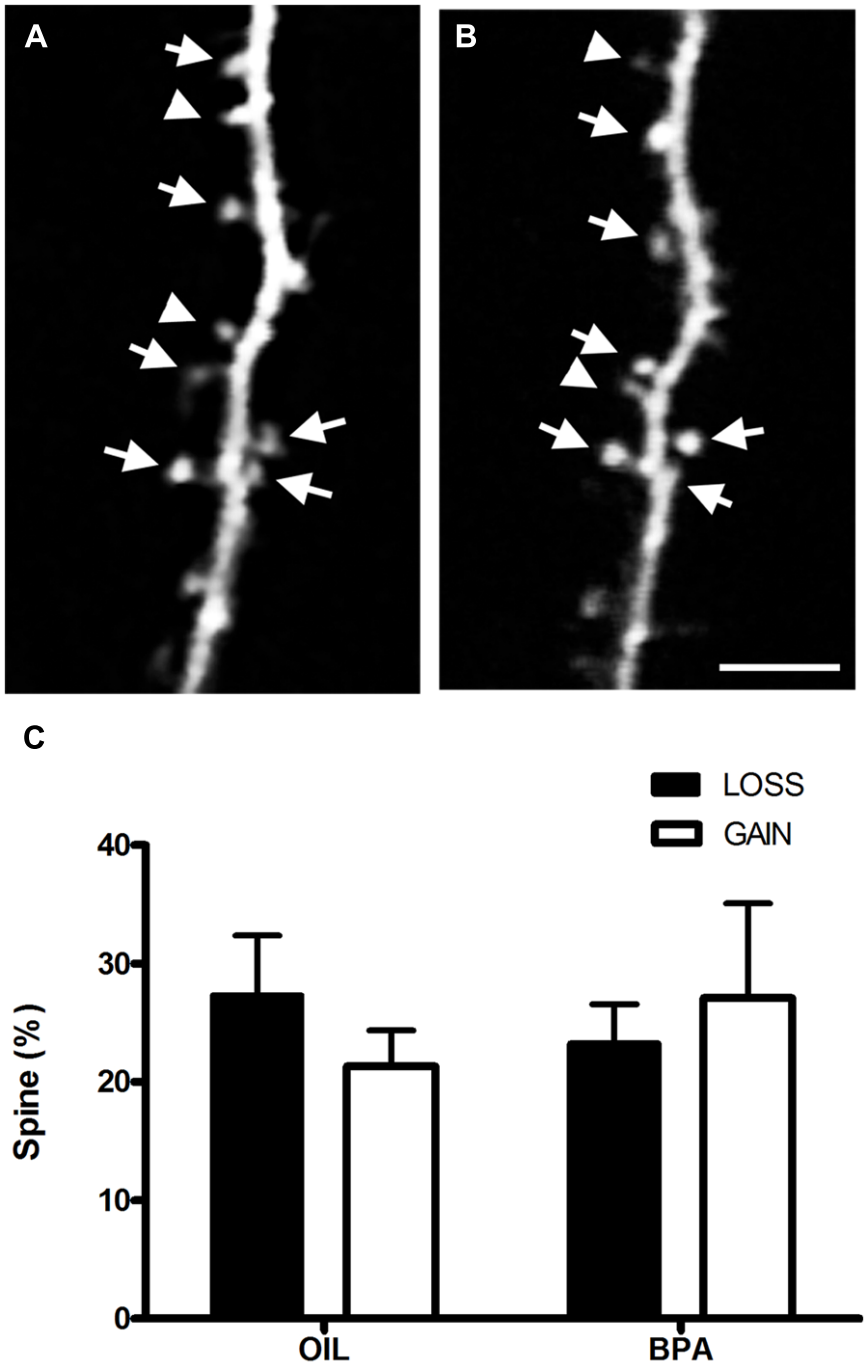
**Bisphenol-A exposure does not alter dendritic spine dynamics.** *In vivo* two-photon images of the same dendritic section taken 4 days apart [Day 0 **(A)**; Day 4 **(B)**] in the somatosensory cortex of a BPA treated mouse. Stable spines are marked with arrows. Arrowheads at Day 0 mark spines that will be eliminated and at Day 4 they mark newly formed spines. **(C)** Quantitative analysis of spine formation and elimination in oil and BPA-treated animals. No significant differences between conditions were observed. Scale bar = 5 μm.

Increased dendritic spine density in BPA treated mice could result from increased formation of dendritic spines or a defect in spine elimination which is prominent during adolescence (Zuo et al., 2005). To determine whether the dynamics of spines were altered we imaged dendritic spines chronically in somatosensory cortex* in vivo* using two photon microscopy (**Figures 6A,B**). We found no change in either formation or elimination rates of dendritic spines over a period of 4 days at P28 (**Figure 6C**), suggesting that increased spine density in BPA-treated animals is established earlier in development and that early BPA treatment affects activity-dependent plasticity without affecting the basal dynamic turnover of dendritic spines during adolescence.

### MICROGLIAL DENSITY IS NOT AFFECTED BY EARLY BPA EXPOSURE

**FIGURE 7:**
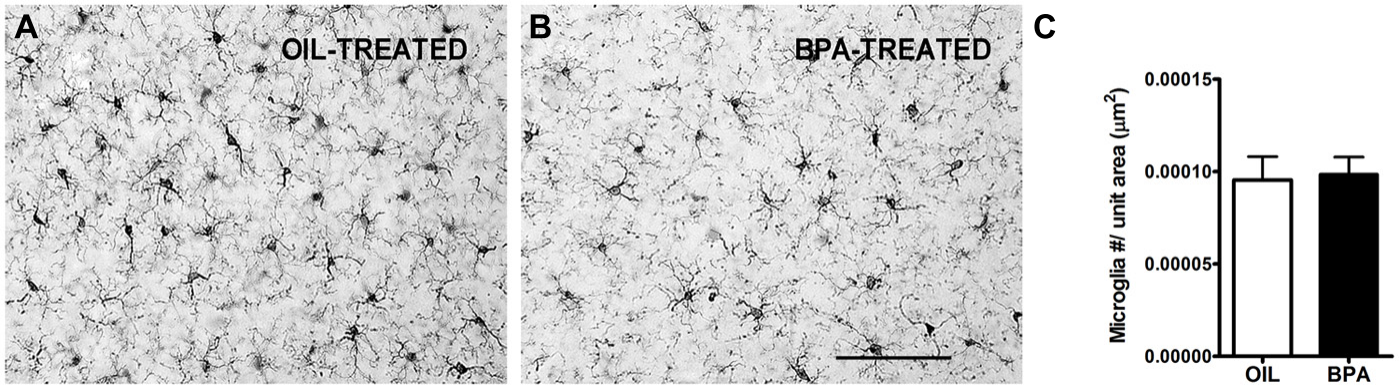
**Microglial density is unchanged in BPA exposed mice.** Representative images showing microglia labeled using Anti-Iba1 immunoreactivity in mice treated with oil **(A)** or low-dose BPA **(B)**. **(C)** Quantitative analysis of Iba1-labeled cell density throughout the cortical layers of visual cortex demonstrates no change in microglial density following BPA exposure. Scale bar = 100 μm.

Given the changes we observed in dendritic spine density, we sought to investigate the potential effects on microglia distribution following low-dose BPA exposure. Microglia are the resident immune cells of the brain and have been implicated in synaptic regulation (Trapp et al., 2007; Perry and O’Connor, 2010; Kettenmann et al., 2013). Previous reports show an effect of low-dose BPA or bisphenol AF (BPAF) exposure on glia, specifically GFAP-positive astrocytes (Yamaguchi et al., 2006; Kunz et al., 2011; Iwasaki et al., 2013) and microglia (Lee et al., 2013). Following low-dose BPA (or oil) exposure, brain tissue was stained with anti-Iba-1, an antibody that specifically labels microglia. Low mag images were collected (**Figures 7A,B**) showing densely labeled cell bodies and fine microglial processes. Quantitative analysis of microglial density was performed in the primary visual cortex. We found no significant difference in microglial density between animals treated with low-dose BPA and animals treated with oil (**Figure 7C**).

**FIGURE 8:**
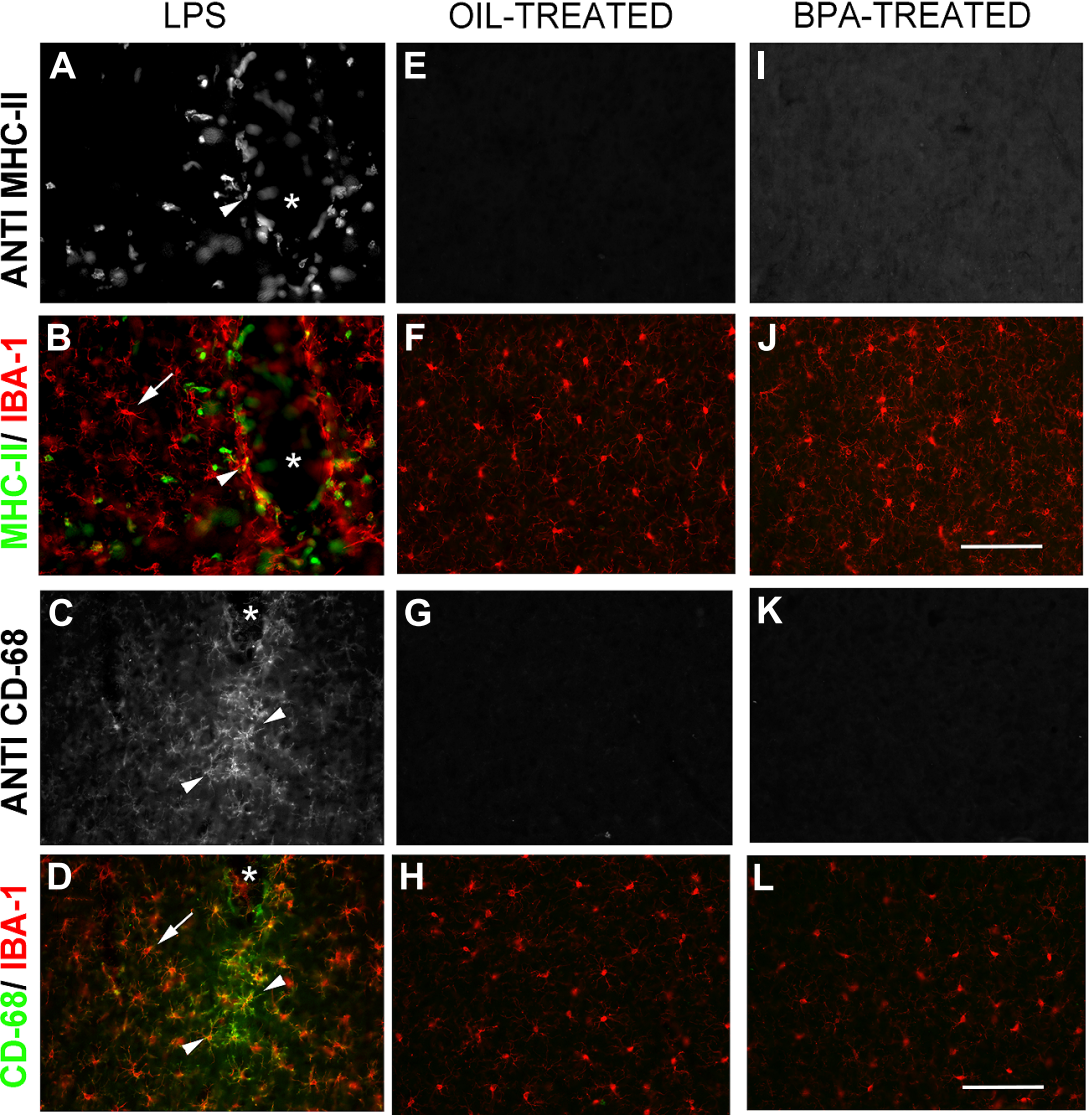
**Bisphenol-A exposure does not result in long lasting microglial activation.** Immunofluorescent reactivity of microglial activation state markers following intracranial LPS injections **(A–D)**, Oil **(E–H)**, and BPA treatment **(I–L)**. Notice that MHC II **(A,B)** and CD68 **(I–L)** are robustly expressed around the injection site in LPS treated tissue and show some co-localization with Iba-1. These markers are absent in oil and BPA-treated tissue. Arrows denote ramified microglia; arrowheads denote co-labeled (activated) microglia. Scale bar = 100 μm.

While both oil and BPA-exposed mice exhibited microglia that appeared to have a resting, ramified morphology, we wanted to determine whether BPA-exposure results in long lasting microglial activation. Using double-immunofluorescent reactivity, we tested the expression pattern of several microglial activation-state markers, including: the cell-surface activation molecule, MHC-II (major histocompatibility complex, type II), and CD68 (a glycoprotein involved in phagocytosis; ED-1). All of these markers were expressed in tissue from animals that had received intracranial injections of LPS, especially around the injection site, where these markers co-localized with Iba-1 (**Figures 8A–D**). In contrast, none of the markers were expressed in oil or BPA-treated tissue (**Figures 8E–L**) suggesting that early BPA exposure does not result in long lasting microglial activation.

## DISCUSSION

The goal of our study was to investigate the consequences of chronic low-dose BPA exposure (one half of the U.S. EPA’s rodent reference dose) during postnatal development (Richter et al., 2007), a critical period when the brain is highly plastic and is thus extremely vulnerable to environmental insults. Exposure during this period has not been well studied despite the fact that childhood is typically the period when human exposures are greatest (Mielke and Gundert-Remy, 2009; Groff, 2010; von Goetz et al., 2010) and also when cortical circuitry is undergoing its greatest experience-dependent refinement. To address this issue, we used MD, a model of neuronal plasticity, which shares many common mechanisms with learning and memory. For binocular mammals reared in a normal visual environment, the majority of neurons in binocular visual cortex respond to stimulation of either eye. If, however, one eye is temporarily deprived of visual input, there is a loss in binocularity and a decrease in the number of cortical neurons in binocular visual cortex responding to vision through that eye. The resulting *OD shift* toward the non-deprived eye has been well documented (Wiesel and Hubel, 1963a,b; Gordon and Stryker, 1996) and is coincident with structural events underlying experience-dependent circuit refinement; including reorganization of cortical circuitry, cell and structural changes, and regulation of important subsets of proteins that regulate synaptic strength (Hensch, 2005). We have shown that mice exposed to low-dose BPA early in development have impaired ODP during the visual critical period when compared to control animals. This effect is not driven by developmental regulation of PV-expressing neurons or MBP levels by BPA exposure and may instead be mediated by altered development of excitatory synapses. The attenuation in ODP was not seen in animals treated within the critical window of visual development (*late exposure)*, suggesting that BPA’s effects on cortical plasticity may be limited to a specific period of early development.

### LONG TERM EFFECTS OF DEVELOPMENTAL BPA EXPOSURE

Experience dependent plasticity of cortical synapses is critically important for functional development and for the remodeling of neuronal networks throughout life. Our finding that BPA exposure can reduce the activity-dependent shift in OD during the visual critical period suggests that BPA can have profound effects on plastic processes within the brain. All the more importantly, ODP plasticity was not induced until 7 days after BPA exposure was discontinued, suggesting that short exposure to BPA during a specific developmental period can have long-lasting effects on the brain. While ODP is a sensory-induced plasticity, similar underlying mechanisms govern learning and memory storage throughout the brain and throughout life (Tropea et al., 2009b; Sur et al., 2013). Additionally, ODP is affected in mouse models of neurodevelopmental disorders (Dolen et al., 2007; Tropea et al., 2009a) suggesting that deficits in ODP may reflect more generalized defects in activity-dependent brain rewiring.

One possible explanation for the effects of BPA on ODP is that BPA may delay or accelerate the development of cortical circuits, making them unable to undergo activity-dependent remodeling during the normal visual critical period (Hensch, 2005) To examine this possibility we investigated two prominent features of cortical development that are known to play an important role in regulating the plastic state of visual cortical circuits.

Inhibition is critical in regulating cortical activity and the excitatory to inhibitory balance is carefully adjusted throughout development and adulthood (Desai et al., 2002). Inhibitory connections generally mature later than their excitatory counterparts (Long et al., 2005), and a specific level of inhibition is permissive for plasticity and opens the critical period for ODP. Conversely, as inhibition continues to mature during the visual critical period, its higher levels inhibit plasticity and close the critical period (Hensch, 2005). Inhibitory neurons, however, are remarkably diverse and show stereotyped and specific connectivity within the cortex. It is becoming clear that PV-expressing neurons are particularly important in opening and closing the visual critical period (Sugiyama et al., 2008; Tropea et al., 2009b). We did not observe a change in PV neuron density in the binocular visual cortex after BPA treatment suggesting that PV cells develop normally and that this pathway is not responsible for the reduction in plasticity elicited by BPA.

Another important determinant of cortical maturity that is inhibitory for plasticity is myelination. Myelination matures during the visual critical period, in a layer specific manner, and limits plasticity by signaling through its receptors which are highly distributed throughout the cortex (McGee et al., 2005). This is analogous to myelin’s role in inhibiting recovery after damage in the CNS (Bregman et al., 1995). However, we also found no evidence of a change in MBP distribution in the visual cortex after BPA treatment, suggesting that myelination is not a target of BPA and does not contribute to deficits in ODP after early BPA treatment.

### THE EFFECT OF BPA ON DENDRITIC SPINES

The lack of changes in important regulators of ODP and cortical maturity, led us to examine the excitatory connections between cortical neurons. Dendritic spines are synaptic compartments whose dynamic behavior is tightly linked to circuit plasticity (Mataga et al., 2004). They receive the synaptic inputs from efferent axons, experience rapid and dramatic changes in density and morphological dynamics during the induction of longer term potentiation and depression (Dunaevsky et al., 1999; Bhatt et al., 2009), as well as during ODP (Hofer et al., 2009). BPA has been found to affect spines, although in a complex manner. Some studies report that administration of BPA alone results in increased spine numbers in the hippocampal CA1 field (Ogiue-Ikeda et al., 2008) while co-administration of BPA and estrogen attenuates increased spine density in rat hippocampus (MacLusky et al., 2005) and prefrontal cortex (Leranth et al., 2008). BPA’s effects on spine density are dependent on timing of exposure (Elsworth et al., 2013) and other studies report decreases in spine density after BPA treatment (Eilam-Stock et al., 2012; Elsworth et al., 2013; Bowman et al., 2014). Our results support the idea that BPA affects dendritic spine morphogenesis, as we observed increased dendritic spine density in the somatosensory cortex of BPA-exposed mice. While we were not able to assess dendritic spines in visual cortex due to the low expression of GFP in this brain area at these early ages, developmental timelines of spinogenesis are similar across primary sensory areas (Elston and Fujita, 2014). The fact that increased spine density after BPA treatment has been reported in different brain areas and different neuronal classes (MacLusky et al., 2005; Xu et al., 2010, 2013b; Eilam-Stock et al., 2012), also suggests that BPA has a widespread effect on the development of brain circuitry. If initial circuit development is altered during the time of BPA exposure, this may affect future plastic processes by altering the excitatory/inhibitory ratio or by altering the flow of information throughout the brain. Additionally, BPA may differentially regulate different periods of dendritic spine and circuit development. Dendritic spine density is known to increase during development during early periods of spinogenesis. Excess and inappropriate connections are then pruned, although the extent of pruning differs between cortical area and between species (Elston et al., 2009, 2010, 2011; Bianchi et al., 2013; Elston and Fujita, 2014). Whether BPA affects synaptogenesis by inducing the outgrowth of inappropriate synapses, or whether it interferes with normal pruning processes that occur during this period of development remains to be explored, although our experiments indicate that BPA does not have a lasting effect on basal spine dynamics.

Increased density of dendritic spines after BPA treatment may reflect altered neuronal activity during cortical development. Early studies showed that visual deprivation decreases spine density in visual cortex and delays the maturation of circuits (Valverde, 1967, 1968). Similar changes occur in the adult somatosensory cortex after deprivation (Kossut, 1998). Thus BPA may alter firing patterns or developmental maturation of cortical pyramidal neurons. It is important to note that we did not observe changes in dendritic spine morphology after BPA treatment. Synaptic morphology is tightly linked with synaptic strength and function (Arellano et al., 2007), suggesting that BPA increases cortical interconnectivity without affecting the maturation and strength of individual synapses. This agrees with our data on PV and MBP expression in the visual cortex and further supports the conclusion that BPA does not delay or accelerate cortical maturation. However, synaptic strength on single neurons is usually tightly regulated by homeostatic processes, whereby if more synapses are made, all synapses are weakened to ensure an optimal level of activity in single neurons (Turrigiano and Nelson, 2004). An increase in synaptic density without a concomitant decrease in synaptic strength may indicate a dysregulation of homeostatic processes by BPA (Xu et al., 2013a,b, 2014). As a caveat, it should be noted that in our study we did not attempt to quantify changes in the complexity of the dendritic tree of pyramidal neurons after BPA treatment, because of the difficulty of getting complete dendritic arbors in thin fixed sections (Elston et al., 2001). Dendritic development is also complex and the way dendritic tree size and branching change with age varies widely between brain areas and species (Benavides-Piccione et al., 2006; Elston et al., 2009, 2010, 2011; Bianchi et al., 2013; Elston and Fujita, 2014). If BPA alters the timing or extent of dendritic outgrowth or pruning, this could greatly change the total number of spines and synapses on each individual neuron in a way that is not directly related to dendritic spine density, and may also have profound effects on the response properties of these neurons (Elston and Fujita, 2014). In fact, BPA has been shown to promote dendritic outgrowth in culture (Xu et al., 2010, 2014). Further studies exploring how BPA affects dendritic arbor development in cortical neurons* in vivo* will be needed to get a full picture of how BPA affects synaptic development in sensory cortices.

### BPA EXPOSURE AND HUMAN HEALTH

Bisphenol-A exposure in humans is generally considered to be lifelong, low and constant, coming mostly from food and beverages but also from the handling of paper receipts, and dust (Vandenberg et al., 2007; Biedermann et al., 2010; Lakind and Naiman, 2011). However, many toxicants are multipotent and can have different effects depending on when they are administered. In this study we attempted to tease out the effects of BPA in a specific developmental period – a time that spans the third trimester and the first years of human life (P5–P21 in mice). This is a period of particular growth in the brain, when neural connections are rapidly made and remodeled. It is also a time when children are at particular risk for BPA exposure through baby bottles and many objects they put in their mouth. We reasoned that this could be a time of particular sensitivity to BPA exposure. Indeed we found a significant decrement in plasticity after BPA exposure during this time period but not acutely, if BPA was administered during the visual critical period when plasticity was assayed. Although it is important to note that the duration and route of exposure, as well as the dose were not uniform in these two experiments, our results may suggest that BPA exposure has its own “critical period” in terms of its long term effects on the activity-dependent remodeling of neuronal circuits, and that the period of late pregnancy and early childhood may be a period of increased sensitivity of the brain to BPA exposure.

The looming question remains of whether effects of BPA exposure observed in rodents can be generalized to humans. The consequences of BPA exposure on human health remain controversial (Vandenberg et al., 2007; Goodman et al., 2009; Beronius et al., 2010). While several studies have reported a negative health impact of exposure in rodents (Howdeshell et al., 1999; Vandenberg et al., 2007; Adewale et al., 2009; Cabaton et al., 2011) others have reported no effect at all (Howdeshell et al., 1999; Tyl et al., 2002, 2008; Ryan et al., 2010). Additionally, experimental variables for the exposure methods – dose, route of administration, diet are factors that widely vary across studies (Thigpen et al., 2007; Vandenberg et al., 2007; Goodman et al., 2009; Myers et al., 2009; Beronius et al., 2010) further challenging extrapolation of rodent studies to human health. It is important to note that in the early exposure paradigm in our study pups received BPA lactationally as only the dams were treated with BPA. The pups therefore received a very small daily amount of BPA because the dams ingested one half of the U.S. EPA’s rodent reference dose, and lactational transfer is limited (Doerge et al., 2010). A number of studies have now suggested that BPA’s effects on the body are non-linear with low doses having disproportionally large effects (Beausoleil et al., 2013; Liang et al., 2014). This further complicates our understanding of how BPA may affect human health but underscores the need to understand the effects of doses smaller than those deemed safe by the EPA.

In this study, we set out to look at the question of whether childhood exposure to daily, low levels of BPA could interfere with normal development of neuronal connectivity. What we found suggests that, in fact, it can; in a profound way; even after exposure has ended. More work is needed to determine the mechanisms by which BPA effects brain development.

## Conflict of Interest Statement

The authors declare that the research was conducted in the absence of any commercial or financial relationships that could be construed as a potential conflict of interest.
